# Foreign Bodies

**Published:** 1889-04

**Authors:** 


FOREIGN BODIES are often removed from the nose in a most tender manner by blowing forcibly through a well-fitting conus into that side of the nose where there is no foreign body.—*Dodd.*

